# Potential Use of Selected Natural Compounds with Anti-Biofilm Activity

**DOI:** 10.3390/ijms26020607

**Published:** 2025-01-13

**Authors:** Dagmara Fydrych, Jagoda Jeziurska, Jana Wełna, Joanna Kwiecińska-Piróg

**Affiliations:** Department of Microbiology, Collegium Medicum of L. Rydygier in Bydgoszcz, Nicolaus Copernicus University in Toruń, 9 M. Skłodowskiej-Curie Street, 85-094 Bydgoszcz, Poland

**Keywords:** biofilm, quercetin, apigenin, arbutin, proanthocyanidin, gallic acid, rutin, vitamin C, phytochemicals

## Abstract

Antibiotic resistance in microorganisms is an escalating global concern, exacerbated by their formation of biofilms, which provide protection through an extracellular matrix and communication via quorum sensing, enhancing their resistance to treatment. This situation has driven the search for alternative approaches, particularly those using natural compounds. This study explores the potential of phytochemicals, such as quercetin, apigenin, arbutin, gallic acid, proanthocyanidins, and rutin, known for their antibacterial properties and ability to inhibit biofilm formation and disrupt mature biofilms. The methods used in this study included a comprehensive review of current literature assessing the bioavailability, distribution, and effective concentrations of these compounds in treating biofilm-associated infections. The results indicate that these phytochemicals exhibit significant antibacterial effects, reduce biofilm’s structural integrity, and inhibit bacterial communication pathways. Moreover, their potential use in combination with existing antibiotics may enhance therapeutic outcomes. The findings support the conclusion that phytochemicals offer promising additions to anti-biofilm strategies and are capable of complementing or replacing conventional treatments, with appropriate therapeutic levels and delivery mechanisms being key to their effectiveness. This insight underscores the need for further research into their clinical applications for treating infections complicated by biofilms.

## 1. Introduction

Plant-derived products have served as effective therapeutic agents against pathogenic microorganisms for thousands of years. In recent years, focus has been placed on the antimicrobial mechanisms of compounds of natural origin.

In the course of the disease, infection with microorganisms that produce a biofilm structure plays an important role [1]. A mature bacterial biofilm is a structural mixture of various planktonic cells, water channels, lipids, polysaccharides, proteins, and extracellular DNA (eDNA). The fundamental component of a biofilm is an extracellular polymeric substance (EPS) referred to as the extracellular matrix (75–90%) and microbial cells [2]. The resulting biofilm is often of a mixed nature. This biofilm can be built by species of microorganisms representing various kingdoms, including protists, fungi, and bacteria. The coexistence of these constantly changing microorganisms is not accidental, as the highly coordinated structure of the biofilm and its physiological complexity demonstrate ecological interdependence [3,4]. A mixed bacterial biofilm formed by several species of microorganisms may be characterized by increased resistance to therapeutic agents due to the interactions of the microorganisms within the biofilm, including metabolic cooperation. Metabolites of one of the microbial species are used as nutrients by the other species forming the biofilm, creating a closed and complementary circulation of matter in the biofilm matrix. This translates into increased formation of biofilm mass in mixed cultures and resistance of microorganisms to external factors, including chemotherapeutics [3,5].

**Figure 1:**
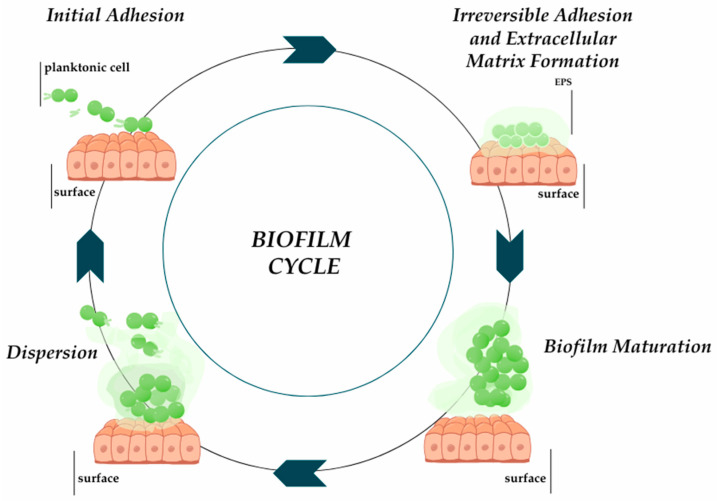
The process of biofilm formation ducts based on [6]. Prepared in Canva^®^ (Sydney, Australia).

The biofilm formation process is influenced by the type and surface properties, and properties of microorganisms. Biofilm formation is complex and takes place in several stages: reversible adhesion, irreversible adhesion, biofilm maturation, and cell dispersion (Figure 1) [6]. The ability of the microorganism to produce biofilm provides protection against dynamically changing environmental conditions [7]. Biofilm has tolerance to antimicrobial agents, which ensures bacterial cells have a greater chance of survival compared to bacteria in planktonic culture [8,9]. Interactions between microorganisms in the biofilm structure, including the exchange of signaling molecules in response to changes in environmental conditions, take place thanks to the quorum sensing (QS) communication system [10]. Tolerance mechanisms also increase the ability to maintain biofilm on various types of surfaces. They colonize the surfaces of medical devices and dead and living tissues [7].

It is estimated that approximately 65% of all bacterial infections are associated with biofilm formation. Biofilm-related infections include urinary tract infections (UTIs); otitis media; endocarditis; osteomyelitis; skin and soft tissue infection (SSTI); dental caries; pneumonia, which is particularly dangerous in people suffering from cystic fibrosis and other lung diseases; and infections associated with the presence of medical devices or implants. Microorganisms can adhere to the surfaces of these medical devices such as joint prostheses, breast implants, mechanical valves and pacemakers, defibrillators, and urinary or venous catheters, with the formation of biofilm on the surface of catheters being described as a common phenomenon. The microorganisms that most frequently contaminate and form a biofilm on the surface of these devices include *Staphylococcus aureus*, *Escherichia coli*, and *Pseudomonas aeruginosa*. Bacterial biofilms can also develop and spread in water distribution systems in health care facilities and elsewhere, and infiltrate food, causing food poisoning and inflammatory diseases of the stomach and intestines [1,6,7].

In recent years, there has been an increase in the spread of Gram-negative bacteria that form a biofilm structure, in which they may have more than 1000 times-reduced sensitivity to antibiotics compared to planktonic bacteria [1,11]. Infection with Gram-negative bacteria capable of producing a biofilm matrix is a particular problem due to the impermeability of the outer membrane and the overexpression of efflux pumps [12,13]. The list of priority pathogens of the World Health Organization (WHO) mainly includes multidrug-resistant Gram-negative bacteria, with particular emphasis on carbapenem-resistant *Enterobacterales* [9]. When choosing a therapeutic strategy, one should look for an antibiotic that is effective against both the biofilm structure and the microorganisms contained within it. The duration and type of antibiotic therapy depend on, among others factors, the site of infection, the severity of the disease, and the properties of the microorganism. Too long a treatment period, the use of broad-spectrum chemotherapeutics before the introduction of targeted therapy, and the unjustified use of carbapenems as the so-called antibiotic of last resort promote the development of antibiotic resistance [14]. It is worth noting that the increasing emergence of resistance to, among others, antibiotics in multidrug-resistant (MDR) Gram-negative bacteria (MDR-GNB) leads to the introduction of the last-chance drug, colistin [15]. Although the use of colistin was considered too clinically toxic in the mid-1970s; numerous adverse effects of polymyxins have been reported, including nephrotoxicity or neurotoxicity [16]. Growing multidrug resistance becomes a challenge when basic resources for the treatment of pathogens are exhausted, especially those that dominate in the hospital environment, which may cause a global epidemiological catastrophe [14]. This indicates a growing need to look for new solutions based on natural and proven principles.

The basic methods of eradicating biofilms include their physical removal and inhibition of regeneration using, among other treatments, antibiotics. Alternative methods of elimination use ultrasound, lasers, and therapy combining light energy with drugs to enhance the anti-biofilm effect. Creating various emulsions or nanoparticles coated with phytochemicals and leading to ferroptosis of bacterial cells is also becoming a popular method. Many plant products have poor or negligible solubility in water, which limits their use. Nanoparticles increase the therapeutic possibilities of plant compounds because they can be designed in such a way as to increase the solubility of the phytochemical. In this combination, a high surface-to-volume ratio is observed for the therapeutic agent with an extended substance release time, which further enhances its antimicrobial potential in clinical use. Antimicrobial medicinal products based on natural products are proving to be more effective than chemically synthesized analogues. Using plant products, you can eliminate bacterial cells present on the surface of the biofilm and inhibit the formation of the biofilm and the proliferation of bacterial cells enclosed in it [16,17,18]. Another method of eliminating bacterial cells is to use the metabolism of iron and reactive oxygen species, resulting in their accumulation leading to so-called ferroptosis. The first stage of introducing phytochemicals into therapeutic strategies is combining them with chemotherapeutics, which would limit the increase in antibiotic resistance. According to scientific reports, combination therapy of a plant product with an antibiotic enhances anti-biofilm activity. It has been proven that plant quercetin, by disturbing the QS system of *P. aeruginosa*, increases the effectiveness of antibiotics used synergistically with it—amikacin and tobramycin. The use of a phytochemical not only increases the exposure of microorganisms to chemotherapeutics but also allows them to be used in lower concentrations, which reduces the occurrence of side effects resulting from their toxicity [12,18].

Many phytochemicals with anti-biofilm activity have been identified in plants. Phyto substances in extracts from plants vary depending on the part of the plant from which the extract was prepared and the time of sampling [17]. One of the plant-delivery substances with anti-biofilm potential is arbutin contained in bearberry leaf extract, which is recommended for treating urinary tract infections, and rutin and ascorbic acid, which are present in most plants [18]. Quercetin, apigenin, gallic acid, and proanthocyanidin (PAC) belonging to the group of flavonoids have several therapeutic properties, including the reduction of free radicals and reactive oxygen species (ROS) at the site of infection. They enhance their accumulation in Gram-negative bacilli, thus disturbing the metabolism, which intensifies the bactericidal effect [17,18,19].

This review aims to present the potential applications of selected natural compounds with anti-biofilm activity and discuss their mechanisms of action against pathogenic microorganisms.

## 2. Quercetin

**Figure 2:**
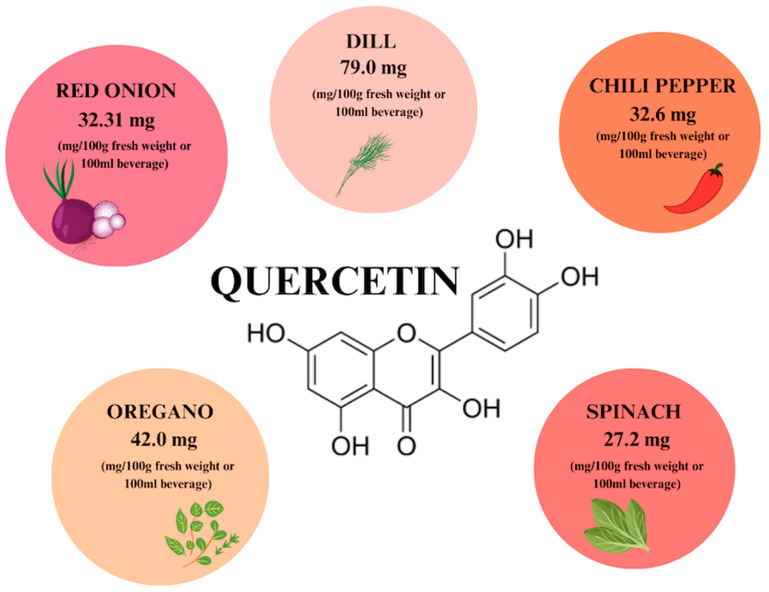
The content of quercetin in individual plant products based on [22]. Prepared in Canva^®^ (Sydney, Australia).

Quercetin (QCT) is a phytocompound commonly found in fruits, vegetables, seeds, and flowers in the form of QCT aglycone, forms conjugated with glycosides and derivatives [20,21]. The content of QCT in individual food products is shown in Figure 2. QCT glucoside conjugates are the dominant phytochemical in the outermost part of the red onion peel, where they constitute 32.21 mg/g of dry weight, which corresponds to 43.6% of the total. This is the form with the highest bioavailability in humans. In addition, high quercetin content is observed in dill (79 mg), oregano (42 mg), and chili pepper (32.6 mg) in 100 g of fresh weight. QCT’s bioavailability can be increased by combining it with vitamin C, folins, and other flavonoids [22,23].

The phytocompound is quickly metabolized in the liver and excreted without accumulating in the body, while pharmacokinetic studies suggest that, after oral administration, it is characterized by low absorption in the free state [23,24]. Phytochemical derivatives, especially glycosides, glucuronide, and sulfate conjugates, are more easily absorbed than the free form, QCT aglycone. The estimated absorption of QCT aglycone after a single oral administration is approximately 2%, while the absorption of QCT glucoside is from 3% to 17% in healthy people receiving 100 mg of the compound. The bioavailability of forms conjugated with flavonoid sugars is influenced by the type of sugar added. 

Available sources indicate that glucosides, which are found mainly in onions and shallots, are much better absorbed than rutinosides, which are present mainly in tea. QCT glucosides are able to interact with glucose transport receptors in the epithelium of the small intestinal mucosa and are then hydrolyzed to the form of aglycone, a large part of which is absorbed in this section. QCT and its derivatives are also converted by intestinal bacteria into phenolic acids and then absorbed and metabolized. The excretion of the ingested phytochemical occurs mainly in the kidneys but also through the respiratory tract [25].

QCT from the flavonoid group has a strong antioxidant effect, which is characteristic of this group of compounds. Its molecular structure is considered the best configuration for binding transition metal ions (including iron, zinc, chromium, and manganese) and capturing free radicals that generate oxidative stress that damages cells. As a flavonoid aglycone, it is soluble in ethanol, methanol, and ethyl acetate and almost insoluble in water [26].

The mechanism of QCT’s antimicrobial action involves damaging the cell wall and changing its permeability. When exposed to the flavonoid, the cell wall is distorted and lysed, which leads to leakage of cytoplasmic content, leading to cell death. Moreover, the plant compound reduces the expression of virulence factors by, among others, inhibition of nucleic acid synthesis, including disruption of the function of the *Escherichia coli* DNA gyrase enzyme, limiting the formation of a biofilm of Gram-negative bacteria. In a complex with iron, QCT intercalates into DNA, causing its significant cleavage into a nicked circular form. The iron–QCT complex also has an anti-adhesive effect, e.g., it binds to a protein ligand FimH of *E. coli* occurring in the urinary tract, which indicates that it may be possible to use quercetin to treat UTIs [26]. A previous experiment involving *P. aeruignosa* strains provided evidence that phytosubstance has the ability to bind with a bacterial single-stranded DNA-binding protein, thereby inhibiting bacterial DNA microorganism synthesis. In *P. aeruginosa*, the plant substance disrupts the QS communication system, reducing biofilm formation, motility, and the production of virulence factors such as protease, elastase, and pyocyanin, even in multidrug-resistant strains [24,25].

In addition to its anti-biofilm effect, QCT has a potential anti-inflammatory and antidiabetic effect, influencing insulin secretion, and also promotes fibroblast proliferation and collagen deposition, which justifies its use in diseases involving SSTI (Table 1) A promising therapeutic strategy using QCT in the treatment of SSTI is coating silver nanoparticles with the compound (Table 2), which eliminates the problem of its solubility, and both products have antimicrobial activity. Silver nanoparticles are an effective agent used in the treatment of wounds, especially burns and diabetics; the addition of QCT increases their therapeutic potential, which provides an avenue for their clinical use and introduction to the pharmaceutical market [25]. The flavonoid also interacts with other biomolecules such as cyclodextrins that are used in the pharmaceutical and food industries. The combination of QCT with water-soluble cyclodextrins increases its bioavailability, which is also a promising solution [27].

## 3. Apigenin 

**Figure 3:**
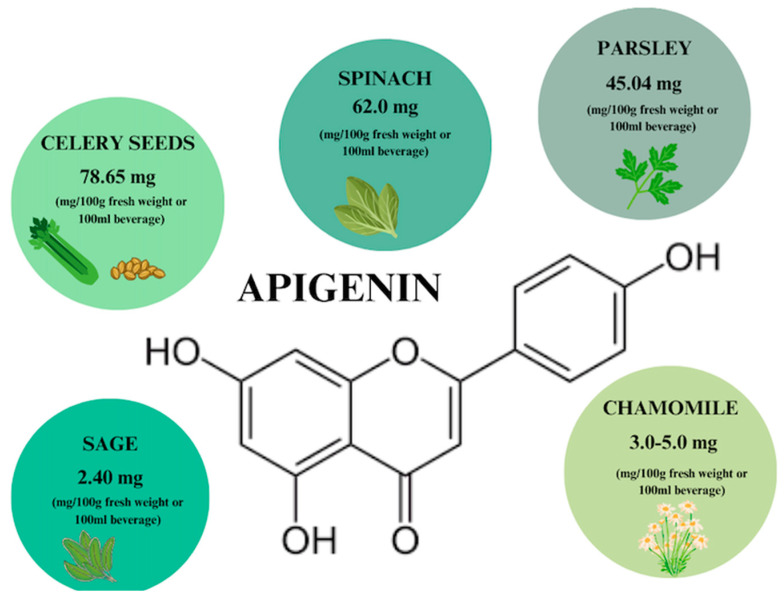
Apigenin content in individual plant products based on [50]. Prepared in Canva^®^ (Sydney, Australia).

Apigenin (AP) is one of the naturally derived compounds found in aglycone and glycoside forms. It is a flavonoid that is widely found as a glycoside in vegetables, fruits, herbs, and plant beverages, such as chamomile infusions [48,49]. According to the literature [50], the highest concentration of AP (78.65 mg) is found in 100 g of celery seeds (Figure 3). 

The flavonoid was classified as Class II according to the Biopharmaceutical Classification System (BCS). This provides information on the poor solubility of the compound in aglycone form, which results in poor absorption and bioavailability. In most studies in rat models, rapid absorption of AP glucosides is found, which is dose-dependent and dependent on the type of food with which it is consumed [51]. The shortest absorption time is characterized by the flavonoid dissolved in dimethyl sulfoxide (DMSO) or ethanol. The glucoside form is more soluble in water than the aglycone; however, it still has poor solubility. To increase this solubility, the aforementioned organic solvents can be used before entering an aqueous solution [52]. 

Pharmacokinetic studies indicate that AP is actively absorbed in the small intestine and, more specifically, in the duodenum and jejunum. The flavonoid is absorbed from solution or food by a passive mechanism in the colon and ileum. It has been shown that 5–10% of AP is absorbed after ingestion of polyphenols [53]. According to literature data, the bioavailability of AP after a single oral administration is 30% [28]. Most intestinal brushtube cells metabolize AP intracellularly, which is supported by extracellular deglycosylation carried out by bacteria found in the colon [53]. The adsorbed AP can undergo intensive metabolism in hepatic phase I, of which the main metabolite is luteolin, and phase II of biotransformation in the hepatic and hepato-intestinal cycle, during which it is conjugated to glucuronide and sulfate conjugates (Table 1). AP is excreted mainly in urine but also in feces, with literature data indicating a higher % excretion in female individuals [54]. AP exhibits a range of nutritional properties and tremendous antioxidant potential. Compared to flavonoids such as QCT, luteolin and kaempferol, AP is the most potent inhibitor of inflammation-induced cyclooxygenase (COX-2) and nitric oxide synthase (iNOS) but also inhibits generated free radicals and Fe^2+^ ions, which it binds, preventing the Fenton reaction, which represents another source of harmful radicals [53]. Free radicals are also caused by tissue inflammation induced by the activity of biofilm-forming microorganisms. In a 2021 study [36], AP contained in the forest fungus *Hydnum repandum* showed antimicrobial and anti-biofilm activity against *P. aeruginosa* and methicillin-resistant *S. aureus* strains. By affecting the change in surface properties, it interferes with microbial adhesion and, thus, biofilm formation. AP also interferes with adhesion by reducing the amount of EPS that maintains biofilm structure, thereby increasing microorganisms’ susceptibility to osmotic pressure and oxidative stress and paving the way for antibiotics [37]. Moreover, it binds receptors for signaling molecules that enable chemical communication, thereby becoming a QS inhibitor. Silencing of communication systems determines the effect on the development cycle and the spread and virulence of biofilm aggregates [10,37,49].

AP was found to be safe and non-toxic even when used in high doses. A promising strategy with AP as with QCT would be the use of nanoparticles (Table 2) for better distribution in the body. Among the studies using a phytosubstance as an active ingredient, hydrogels and creams with potential use in the treatment of skin and deeper tissue infections are promising. They show simultaneous promotion of the formation of blood vessels and their contraction, preventing water loss. The flavonoid enhances collagen synthesis and deposition, which is another property that promotes regeneration and justifies its use in plastic surgery as a component of skin flaps; it is also possible to use AP in diabetic wound treatment. The properties of the phytochemical are also desirable for treating UTIs; these properties include a diuretic effect which, while inhibiting the adhesion and proliferation of microorganisms to the epithelium of the urinary tract, results in their flushing out upon urination [37]. Due to a number of beneficial properties resulting from the use of AP, it is worthwhile to conduct research on new therapeutic agents that would contain this phytochemical in their composition. Another advantage is the fact that the compound is found in natural products that are available to the public, which creates the possibility of obtaining the substance by alternative means, e.g., from agricultural waste, which would also be beneficial to the environment, as opposed to the production and disposal of chemotherapeutics [48].

## 4. Arbutin

**Figure 4:**
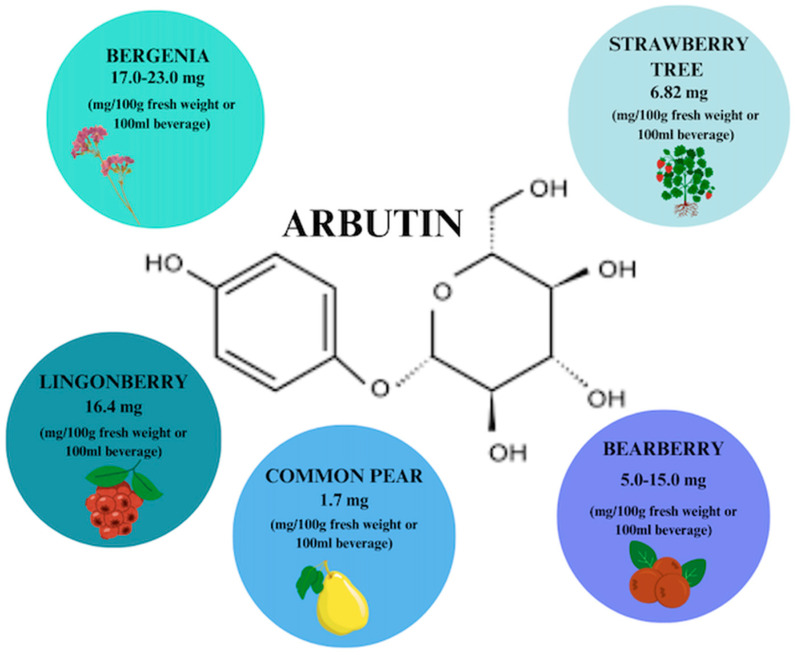
Arbutin content in individual plant products based on [56]. Prepared in Canva^®^ (Sydney, Australia).

Arbutin (ARB) is a phenolic hydroquinone glycoside that can be found in fruits, roots, and honeys, but is primarily found in leaves. The highest concentration of phytocompound, i.e., about 17.0–23.0 mg/100 g fresh weight, is found in thick-leaved Bergenia [55]. It exists in two isomeric varieties with α-ARB being the synthetic variety used to lighten the skin while β-ARB is the form naturally extracted from leaf extracts of, e.g., bearberry (Figure 4) used to treat lower UTIs [18,56]. Naturally occurring ARB can also be used in place of α-form, which raises the possibility that it can also be used to treat skin diseases, facilitating skin regeneration and wound scar care. The efficacy of the extracts in the treatment of UTIs has been confirmed and they have been deemed safe by the European Medicines Agency (EMA) [56].

After oral administration, β-ARB is absorbed in the small intestine, undergoing hydrolytic conversion to hydroquinone and glucose in the liver. Hydroquinone is conjugated with glucuronic acid and sulfuric acid and is excreted in this form by the kidneys. In the case of lower UTI, the conjugated substance is hydrolyzed in the urinary tract and the resulting hydroquinone exhibits antimicrobial activity. The mechanism of action of the plant metabolite is based on the destruction of the bacterial cell wall, resulting in the leakage of intracellular contents and cell death [55,56]

ARB is a glycoside used in the treatment of UTIs of bacterial etiology, such as *E. coli* and *P. aeruginosa*, and fungal etiology, such as *Candida albicans* [38]. Its activity is associated with changes in the structure and function of bacterial cells and their biofilms through inhibition of gene expression and disruption of QS. Moreover, using the phytochemical in combination with birch sap reduces the frequency of recurrent bladder infections. Prior to the use of ARB, the only obstacle related to its safety in terms of the effects on the kidneys, while nephrotoxicity of the compound was ruled out, with no side effects found in terms of kidney function, lymphocytes, or disruption of human DNA integrity [38,56].

The phytochemical exhibits tremendous antimicrobial potential and has anti-inflammatory effects due to inhibition of the Akt immunosuppressive pathway, and it is used to treat diabetes due to its ability to lower glycemic levels. β-ARB is already used as an ingredient in an adjunctive preparation for UTIs called Ixtasol© with proven bactericidal, bacteriostatic, and anti-inflammatory properties and without side effects, accompanying synthetic antibiotics such as nephrotoxicity and generation of antibiotic resistance [38].

## 5. Proanthocyanidin

**Figure 5:**
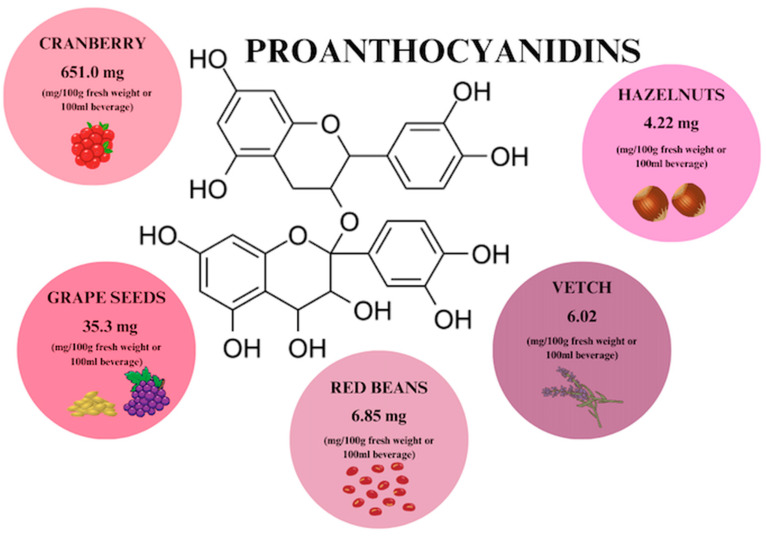
Proanthocyanidin content in individual plant products based on [29,58,61]. Prepared in Canva^®^ (Sydney, Australia).

Proanthocyanidins (PACs) are a group of polyphenols that are naturally, ubiquitously found in many vegetables, plant skins, seeds, flowers, fruits, and nuts (Figure 5), [29,57]. The richest source of PACs is cranberry extracts, which are recommended as a treatment for what is considered to be the world’s most common infectious disease—UTI [19]. PAC constitute 71% of the total content of phenols in cranberry and their content in the Ben Lear cranberry fruit variety is 651 mg/100 g [19]. PACs are composed of flavan-3-ol subunits of epicatechin, catechin, gallocatechin, or epigallocatechin. There are C4–C6 and C4–C8 double bonds between the chains. Structural complexity is influenced by the type of polymerization, which is a direct consequence of bioavailability [19,57,58]. The highest bioavailability is characterized by proanthocyanidin dimers, more precisely epicatechin, with an absorption rate of 5–10% [57]. Oligomeric proanthocyanidins (OPCs) include PACs with a lower degree of polymerization (2–4 monomers), while molecules containing more than 5 monomers are called polymeric proanthocyanidins (PPCs) [59,60].

In the liver, there is an increase in the formation of monomer conjugates, which are then released into the circulation. This is followed by excretion in urine, accumulation in tissues, or particulate return to the intestine with bile via the hepato-intestinal circulation [59]. It has been shown that monomers with a degree of polymerization above four are absorbed in the large intestine, due to the fact that high-monomer polyphenols are metabolized by intestinal microflora [60].

Grape-derived PACs exhibit anti-inflammatory effects by reducing the accumulation of pro-inflammatory cytokines, but also influence the transformation of muscle fibers, as a result of which they retain more water, are more resistant to fatigue, and reduce the content of saturated fatty acids. The use of PACs may reduce the risk of chronic diseases, including obesity, atherosclerosis, and hypertension, which increase the risk of myocardial infarction. Additionally, PACs from grape seeds have the potential to limit the growth and development of both anaerobic and aerobic microorganisms, e.g., on the surface of plaque [1,62,63]. In a 2019 study [41], PACs derived from cranberry extracts were proven to not only inhibit biofilm formation but also prevent the acquisition of tetracycline resistance in *P. aeruginosa* and *E. coli*.

Inhibition of the biofilm formation also takes place by disrupting the communication system, both by PAC and by combining the phytochemical with the antibiotic ciprofloxacin. It is worth mentioning that PAC-containing extracts affect both actively metabolizing cells and those that are dormant in the biofilm structure by exposing them to antibiotics [41]. PACs, similarly to apigenin, show anti-adhesive properties against bacteria (Table 1 and Table 2) found on the surface of the epithelium of the urinary tract and intestines like *E. coli*, which reduce their virulence [1]. Plant compounds are able to reduce the stiffness and adhesion strength of the bacterial cell envelope, which indirectly inhibits biofilm formation. Microorganisms influenced by PAC both in the form of planktonic cells and enclosed in the biofilm matrix are characterized by morphological changes such as abnormal shape, and a change in membrane potential and disruption of the membrane integrity of bacterial cells are noted, which delays their growth [64]. The delay also applies to the motility of the microbial swarm, which was confirmed to act against *P. aeruginosa*. In the course of this study, a reduction in biofilm formation of half the initial value was achieved using a rather low concentration of PACs (1 µg/mL) derived from cranberry juice. Moreover, in PACs-treated *P. aeruginosa* strains, a QS agent was found, which is another mechanism of biofilm inhibition [64].

## 6. Vitamin C 

**Figure 6:**
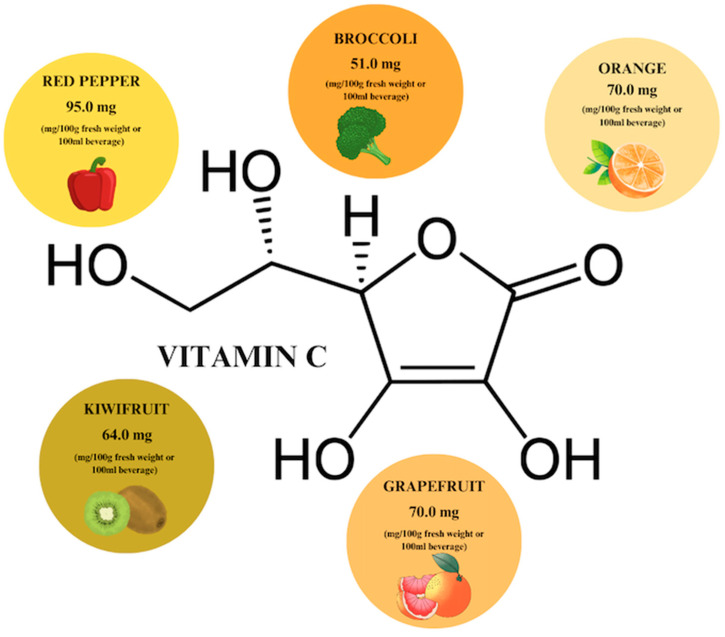
Vitamin C content in individual plant products based on [65]. Prepared in Canva^®^ (Sydney, Australia).

Vitamin C is the most popular phytochemical, which is especially renowned for its antioxidant activity. It can be extracted from citrus fruits, berries, tomatoes, and green leafy vegetables. The highest content of vitamin C (VC) is found in red pepper (95 mg/100 g), orange, and grapefruit, which contain equal concentrations of the phytocompound (70 mg/100 g) (Figure 6) [30,65]. When using VC, also known as ascorbic acid, it is important to keep in mind the reference values for daily requirements, i.e., 110 mg/day for men and 95 mg/day for women, set by the European Food Safety Authority [66]. Further increasing the concentration of the plant compound reduces its absorption in the intestine, which is important information to consider in relation to supplementation (Table 1).

The most bioavailable form of VC is L-ascorbic acid. In vivo, it occurs in two forms: ascorbate (ASC, reduced form) and dehydroascorbic acid (DHA, oxidized form), with the reduced form predominating. The bioavailability at doses up to 200 mg/day is 70–90% of the absorbed compound. VC is water-soluble and its absorption takes place both through the oral route and through the skin, involving three types of membrane transport: passive diffusion, facilitated diffusion, and active transport. For the most part, the absorption, metabolism, and excretion of the phytochemical is regulated by a family of sodium-dependent VC transporters (SVCTs), whose action is influenced by the dose of the ingested compound. Orally ingested ascorbic acid is absorbed by enterocytes mainly in the form of ASC via sodium-coupled transport or as DHA via diffusion facilitated by glucose transporters 1 and 3. DHA is converted to ASC and, in this form, is transported into the bloodstream to localize intracellularly. ASC is efficiently filtered by the glomeruli and excreted in the urine after being biotransformed to DHA and then to sulfate and oxalic acid [42,67].

ASC plays an important role in maintaining the oxidative-reductive state of cells, but is also involved in the epigenetic control of gene expression by increasing the activity of Fe^2+^-dependent dioxygenases. Thus, it indirectly participates in DNA demethylation and enhances iron absorption through the reduction of Fe^3+^, the form most commonly supplied from food, to the Fe^2+^ form, which increases its intestinal absorption. This activity is also a highly useful cofactor in collagen biosynthesis, which makes the phytochemical helpful for skin regeneration after infection has subsided [65].

According to [42], in which biofilms were subjected to the effects of ascorbic acid, the plant compound showed 100% inhibition of *P. aeruginosa*, with the test strains also showing reduced expression of the genes responsible for biomass formation and antibiotic resistance. Similar effects were obtained by treating carbapenem-resistant *Klebsiella pneumoniae* strains with VC. This indicates that the use of VC in upper respiratory tract infections or infections related to the respiratory tract, such as cystic fibrosis, is an element supporting the therapy. The antimicrobial activity of ascorbic acid is based on pro-oxidant activity and subsequent production of ROS, which accumulate in the cells of biofilm-forming microorganisms. As a consequence, lipid ultra-oxidation, redox imbalance, and DNA damage occur, inhibiting bacterial growth. In multidrug-resistant strains treated with VC at a concentration of 8 mg/mL, a reduction in the production of biofilm matrix polysaccharide is observed, which also inhibits biofilm formation and development. One of the elements necessary for biofilm function and increasing microbial resistance to chemotherapeutics are efflux transporters (efflux pumps), whose activity also decreases after VC is introduced into cells [42,68]. Prophylactic VC supplementation can prevent infection with a uropathogenic *E. coli* strain leading to a UTI, as the compound acidifies urine, which is poorly tolerated by microorganisms [68].

Ascorbic acid has many beneficial properties, including research-confirmed [42] anti-biofilm activity, while being a readily available, inexpensive compound with virtually no side effects. It has also been shown to increase the activity of antibiotics such as piperacillin, piperacillin/tazobactam, ceftazidime, ciprofloxacin, and gentamicin when used synergistically with VC, making the compound a potential modifier of antibiotic resistance. Further in vivo studies on the antimicrobial activity of ascorbic acid are needed; however, it is definitely worth supplementing individually or in combination with antibiotics to shorten the duration of treatment [66,68].

## 7. Gallic Acid

**Figure 7:**
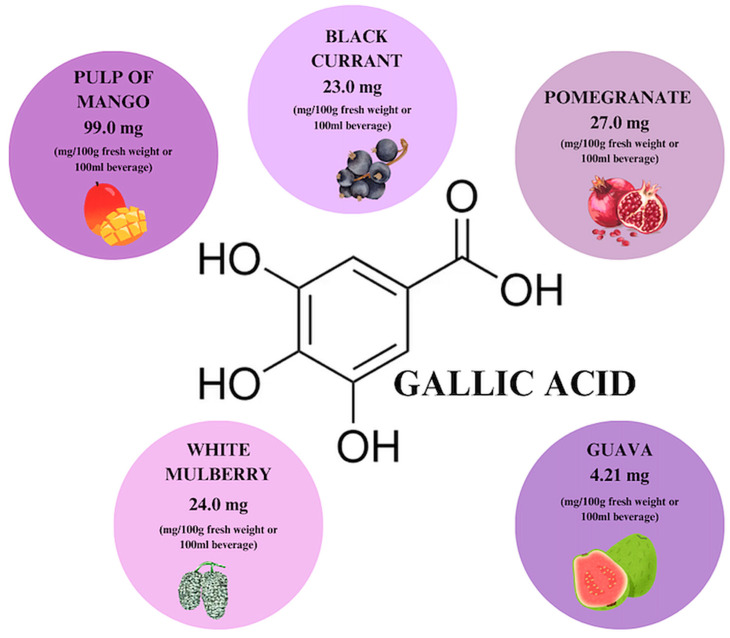
Gallic acid content in individual plant products based on [69]. Prepared in Canva^®^ (Sydney, Australia).

Gallic acid is a phenolic acid that is found in almost every part of many plants, including their leaves, fruits, roots, and seeds (Figure 7). Its highest concentration is observed in mango pulp (95–99 mg/100 g dry weight) and blackcurrant leaves [31,69]. It occurs both in the form of free gallic acid (GA), dimers of GA–ellagic acid, and as a secondary metabolite formed during the hydrolysis of gallotannins. During the decomposition of GA, among other things, ester derivatives of the compound are formed, with epigallocatechin gallate (EGCG) indicated as the main bioactive substance among them [31].

Pharmacokinetic studies of the phytochemical suggest that the absorption and elimination of GA after oral administration is rapid, with dosage adjustment favorably increasing its bioavailability (Table 1). When used per os, the bioavailability of the phytochemical is determined to be 70%. Moreover, no side effects due to its toxicity were found in animal studies [31,70]. At the same time, it is worth noting the favorable application and research solubility of the plant compound, since gallic acid has good solubility in water—when conducting experiments, this eliminates the need to check the effect of the solvent on the biofilm beyond the influence of the tested compound [34]. 

Polyphenols, which include gallic acid, are rapidly absorbed in the gastrointestinal tract, characterized by rapid metabolism in human intestinal cells and a high degree of elimination in vivo. The main metabolite of plant phenol in human plasma and urine is 4-O-methyl gallic acid (4-OMeGA)—¾ of the absorbed compound is excreted in this form by the kidneys, while about 20% is excreted unchanged. Tandem mass spectrometry (MS/MS) analysis after a single oral administration showed that the phytochemical was distributed mainly in the kidneys, while the second-highest GA content was found in lung tissue [31]. 

GA shows antimicrobial but also anti-inflammatory properties, which justifies its therapeutic effect on gastric mucosa damaged by non-steroidal anti-inflammatory drugs. It prevents oxidative stress and inhibits the activation of the mitochondrial apoptosis pathway. Studies have noted its antiproliferative effect in the context of gastric adenocarcinoma cells, as well as its protective effect against stomach cells against ethanol-induced gastric ulcers, which is also attributed to its antioxidant properties and inhibition of apoptosis. With regard to skin and subcutaneous tissue infections, it is worth mentioning that the phytochemical enhances the process of tissue regeneration by accelerating the migration of cells involved in skin formation and activates factors specific to this process, such as adhesion kinases [34,70].

The use of GA in antimicrobial therapy is justified because of its proven inhibitory effect on bacterial adhesion and delayed EPS formation. It also modifies the permeability of the microbial cytoplasmic membrane and quiets QS by reducing acyl homoserine lactones (AHLs) synthesis. Phenolic acid disrupts bacterial ion homeostasis by causing leakage of potassium ions, thereby promoting cell death. It has the ability to chelate calcium ions that are necessary for growth and enzyme activity. By affecting the function of bacterial proteins, it reduces the iron uptake capacity and, consequently, the formation of EPS by *P. aeruginosa* and *P. mirabilis*. The production of siderophores that capture this element is, together with QS, an element of biofilm formation that further determines the antibiotic resistance profile of strains. By closing off microbial access to iron, GA becomes an important eradication compound. Another mechanism through which phenolic acids facilitate bacterial elimination is competition with AHLs for binding to receptors. The consequence is a reduction in the expression levels of the lasR and lasI genes, which are important regulators of the QS system. Inhibition or mutation of the lasR regulatory gene is therefore another potential therapeutic strategy [10,71]. 

Promising therapeutic agents containing GA are hydrogels (Table 2), in which the phenolic acid occurs in combination with thermosensitive chitosan, providing high strength and adhesion. Such dressings are used for healing skin injuries, promoting wound closure and regeneration of inflamed tissues [72].

The rapid metabolism and elimination of gallic acid can result in an ambiguous degree of bioavailability after per os administration; however, great opportunities are provided by the still-developing colloidal and phospholipid therapeutic strategies, thus eliminating this deficiency. An increase in bioavailability and, thus, effectiveness can be achieved by combining the phytochemical with soy lecithin or through a synergistic mechanism when administered together with other phytochemicals such as catechins present in the cocoa pulp. The observed enhancement of antimicrobial effects confirms the efficacy of such combinations [34,44,70].

## 8. Rutin

**Figure 8:**
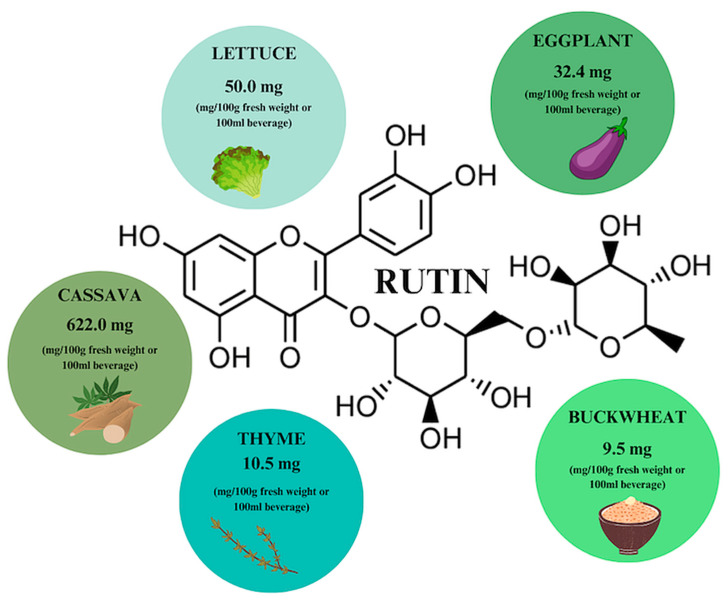
Rutin content in individual plant products based on [17]. Prepared in Canva^®^ (Sydney, Australia).

Rutin is a flavonoid that is abundant in our diet, and especially in plants such as common rue—the first plant in which rutoside, otherwise known as sophorin, vitamin P, and quercetin-3-O-rutinoside, has been detected. The sources with the highest rutin (RT) content include cassava, eggplant and lettuce, with the highest RT concentration observed in winter-harvested lettuce (Figure 8) [17,32]. The phytochemical is characterized by low solubility in water—organic solvents like ethanol or DMSO would be a better choice; however, it is characterized by high stability during temperature elevation. When buckwheat is heated to 150 degrees, an increase in RT content is achieved, while the opposite effect is achieved when the plants are processed, e.g., bread baking. Buckwheat contains 9.5 mg/g dry weight of RT, while bread contains 0.24 mg/g. It is also possible to enzymatically convert RT to QCT by adding water and yeast [17,32,33].

Pure RT has a bioavailability of about 20% after oral ingestion. As QCT glucoside, it is transported via glucose transporters in the intestinal mucosa and then, like QCT, metabolized and absorbed in a form devoid of the sugar residue. The metabolism of the absorbed compound is aided by the intestinal microflora, which converts RT derivatives to phenolic acids, which are also absorbed and excreted mainly by the kidneys and lungs, as is the case with QCT, which was mentioned earlier [32].

As a phenolic compound, RT has antioxidant properties, as evidenced by the results of a study on a preparation containing the phytochemical, which exhibited an increase in free radical elimination by 40% compared to preparations lacking in the plant substance. Moreover, the substance shows photoprotective effects and is used as an ingredient in cosmetics for sun protection by cult brands such as Bielenda or Cerave, for example, which also proves the clinical safety of the ingredient [33]. One can also find studies attesting to the potential of RT in the treatment of Alzheimer’s disease in a rat model, in which it showed neuroprotective effects by counteracting lipid peroxidation, among other things. Maintaining low levels of lipids, glucose and lactate in response to the pathogen along with promoting phagocytic activity of macrophages demonstrates the immunomodulatory capacity of the phytochemical [32,33]. 

RT shows therapeutic potential for bacterial infections (Table 2). It has the ability to inhibit the growth of *E. coli*, *Pseudomonas vulgaris*, *Shigella sonnei*, *Klebsiella* spp., *P. aeruginosa*, and *Bacillus subtilis*, which is associated with inhibition of bacterial DNA isomerase [33]. In a study by Qaralleh H. [47], a reduction in the production of pyrocyanin, rhamnolipid, and the activity of enzymes such as proteases and chitinases was obtained in strains of *P. aeruginosa* with an extended-spectrum beta-lactamase (ESBL) resistance profile using a methanolic extract containing RT already at concentrations < 3 mg/mL. Inhibition of bacterial biofilm formation was observed by decreasing aggregation, hydrophobicity, motility, and EPS production under the influence of a phytosubstance. Inhibition of biofilm development was also achieved through genetic suppression; decreasing pslA gene expression significantly decreased AHL secretion, which directly interfered with the QS system. Obstacles to the use of phytochemicals as part of an antimicrobial therapy strategy are aspects of bioavailability, which are no longer a significant problem nowadays due to the existence of many methods that allow even lipophilic substances to be delivered to target cells. In the case of RT, it is possible to use an encapsulation technique that allows controlled release under gastrointestinal conditions and increases the solubility of the compounds. The most popular design structures are nanometer-sized particles (Table 2), hydroxypropyl methylcellulose (HPMC) associated with colloidal systems, e.g., RT-containing nanoemulsions, in which a 25-fold increase in solubility and bioavailability of the drug after oral administration is achieved [73]. Another promising therapeutic strategy is hydrogels on xanthan gum and hydroxypropylmethylcellulose loaded with complexes of RT with 2-hydroxypropyl-β-cyclodextrin—this combination allows controlled fixation of the phytochemical over a longer period of time to improve its bioavailability, which is achieved by swelling of the hydrogels with simultaneous release of RT from the complex. The hydrogels positively inhibited the growth of both Gram-positive and Gram-negative bacteria, which demonstrates their high antimicrobial potential. Its hydrogel formula and anti-biofilm properties make RT a promising solution in wound-healing applications, for example, in the treatment of diabetic wounds [47]. The development of drug delivery technologies offers great opportunities for the use of plant-derived compounds as potential antimicrobial preparations, whose undoubted potential, confirmed by numerous studies, has previously remained untapped due to the pharmacokinetic limitations of the compounds.

## 9. Discussion

Compounds of plant origin show great potential in eradicating developing and mature biofilm and also support the action of antibiotics, and thus they have come to be considered as alternative methods of combating biofilm-related infections. In addition to their antimicrobial properties, they have properties that enable their use in the treatment and care of wounds and tissue defects, as they prevent the enzymatic degradation of collagen while stabilizing it, have a beneficial effect on the functioning of blood vessels, and have anti-inflammatory effects by reducing oxidative stress and modulating the activity of inflammatory mediators, which is desirable in various treatments [11,52]. The enormous therapeutic potential of phytochemicals is subject to certain limitations, such as their low bioavailability, which is associated with poor solubility in water and fats, and which results in poor penetration into target cells. Nowadays, this is not such a significant problem due to the continuous development of modern drug delivery systems, thanks to which it is possible to increase their absorption and biological activity in target tissues. The use of compounds of plant origin or plant extracts associated with nanoparticles or nanoemulsions contributes to increasing the therapeutic potential of the plant extracts and their ingredients by improving their stability and solubility, prolonging their release, and targeting the site of action. In the future, modern drug delivery systems will enable an increase in the use of phytochemicals in the pharmaceutical industry to provide an alternative effective therapy [1]. Nanoparticle systems for the targeted delivery of both hydrophobic and hydrophilic substances are of great interest. The gold standard in the healing of wounds, especially diabetic and burn wounds, are silver nanoparticles (AgNPs), in which plant substances such as quercetin can be bound to increase the anti-biofilm effect. This complex not only accelerates regeneration but also facilitates the elimination of microorganisms trapped in the biofilm matrix. When used antimicrobially, AgNPs are distinguished by strong broad-spectrum activity and reduced generation of resistance, and their effectiveness is additionally increased by synergistic action with antibiotics such as ciprofloxacin and tetracyclines [27,35]. Another example are nanoemulsions containing rutin (NE-RU), which increased stability and bioavailability by 34 times after oral administration [73].

Flavonoids have great potential in the treatment of skin infections, urinary tract infections, and other diseases related to the presence of microorganisms that produce a biofilm structure. The microorganisms most frequently isolated from biofilm-related infections are Gram-positive cocci, such as *S. aureus*, and Gram-negative bacilli, including *P. aeruginosa* and *E. coli*. As anti-biofilm molecules, compounds derived from plants can act on biofilm components, inhibiting their development or eliminating it. One of the ways to limit the functioning of the biofilm is to block QS communication, which contributes to the weakening of bacterial virulence by reducing the adhesion ability, motility, and production of exotoxins and cytotoxins, as well as elastase and protease in *P. aeruginosa*. By lowering the activity of bacterial toxins and enzymes, it is possible to reduce the harmful activity of the microorganism in SSTI, pneumonia, keratitis, and sepsis [1,52]. This effect is attributed to garlic extract, which is rich in QCT, GA, and RT. A plant compound that significantly reduces the adhesion of *E. coli* is PAC extracted from cranberries. Cranberry PACs are able to competitively bind the fimbriae of P-type bacteria and deform them, preventing them from adhering to the epithelium of the urinary tract and intestines. This may limit the colonization of pathogenic *E. coli* strains responsible for urinary tract infections and food poisoning [19].

β-arbutin, contained in a preparation supporting the treatment of urinary tract infetions called Itxasol©, also has the potential to eradicate UTIs [38]. Biofilm-inhibiting *S. aureus* has VC, which, thanks to its high oxidative potential, generates oxidative stress and the formation of reactive oxygen species. They are directed to bacterial cells, leading to disturbances in their structure and metabolism, facilitating the eradication of cocci biofilm from wounds and the oral cavity [40]. Apigenin contained in the extract from the edible mushroom Hydnum repandum has a negative effect on the biofilm of *S. aureus*, *E. coli*, and *P. aeruginosa*. A synergistic effect with antibiotics has also been confirmed, which allows us to conclude that the phytocompound prevents the development of resistance to chemotherapeutics in bacteria [41]. Phytochemicals can be an auxiliary treatment during antibiotic therapy or can act as a replacement for antibiotics. They are effective against both planktonic forms of microorganisms and those enclosed in the biofilm matrix. To inhibit the biofilm formed by several species of bacteria, a higher concentration of the phytochemical should be used than that used for microorganisms occurring in monoculture [42]. Microorganisms found in biofilm have increased tolerance to antibiotics. Phytochemicals prove effective against them when used individually and can also support the action of therapeutic agents by acting synergistically. The increasing frequency of isolation of methicillin-resistant *S. aureus* (MRSA) strains poses a serious threat, since they are difficult to treat but easily spread, especially in the hospital environment. According to research, of the 622,390 infections caused by multidrug-resistant bacteria, 52% of them are MRSA infections. It has been proven that *Krameria lappacea* root extracts rich in proanthocyanidins have eradication properties against both planktonic forms of MRSA and those protected by biofilm, inhibiting growth and inducing the death of bacterial cells. Moreover, in naturally occurring doses, the extract does not show cellular cytotoxicity, which also supports its therapeutic potential [40]. Proanthocyanidins isolated from *K. lappacea* are able to inhibit the growth of all *S. aureus* MRSA strains when used at the same concentration as ciprofloxacin. Proanthocyanidins are natural compounds to which microorganisms have not developed a resistance mechanism [40]. The purified fraction of cranberry proanthocyanidins also enhances the activity of a wide range of classes of antibiotics against *E. coli* and *P. aeruginosa*. Tetracycline resistance in these microorganisms is often the result of evolutionarily acquired resistance. It has been shown that when tetracyclines are used against these Gram-negative strains in combination with PAC, it is possible to completely inhibit the development of resistance to this class of therapeutics. What is more, such therapy may potentially reduce the spread of existing resistance and prolong the effectiveness of currently available therapeutic agents. Such supported therapy limits the development of bacterial biofilm and has an effect on both actively metabolizing cells and those dormant cells exposed to antibiotics [40,41]. It has been shown that PACs also enhance the in vitro and in vivo activity of sulfonamides against *P. aeruginosa* and *E. coli*, increase bacterial tolerance to tetracyclines, and are even able to eliminate natural resistance to this group of antibiotics [41]. VC is also distinguished by its ability to inhibit the biofilm of uropathogenic MDR *E. coli* strains, and exhibits both individual and synergistic effects with antibiotics. MDR strains are resistant to cephalosporins, carbapenems and fluoroquinolones. In a previous study, the phytosubstance caused biofilm inhibition in 43% of the tested strains qualifying as non-biofilm producers while reducing the expression of biofilm-related genes. VC shows synergistic activity with most of the antibiotics tested, i.e., levofloxacin, meropenem, ceftazidime, and nitrofurantoin [43].

**Figure 9:**
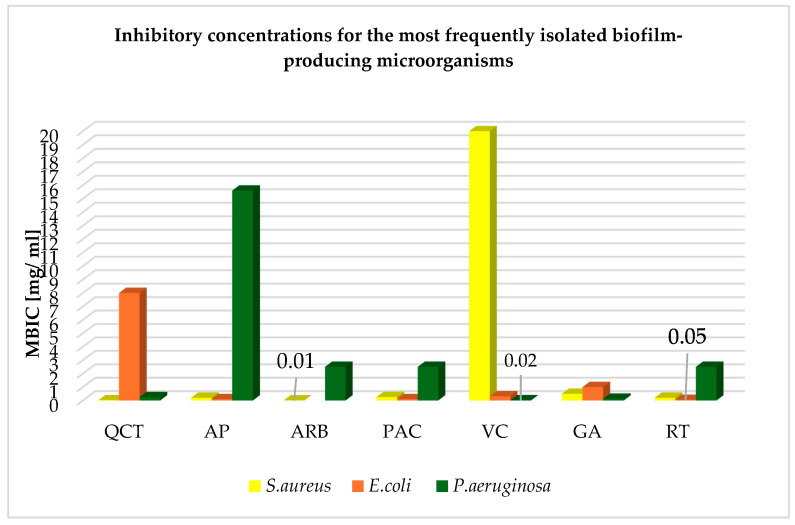
Inhibitory concentrations of phytochemicals for the most frequently isolated biofilm-producing microorganisms based on Table 2.

The inhibitory concentrations of the mentioned phytochemicals and their forms, i.e., QCT, AP, ARB, PAC, VC, GA, and RT, against the most frequently isolated biofilm-producing microorganisms are presented in Table 2. The table includes the minimum inhibitory concentrations (MICs) for planktonic cells of microorganisms and the minimum biofilm inhibitory concentration (MBIC) found in the literature, along with the specificity of the strains tested, i.e., the mechanism of resistance and its occurrence in a mixed biofilm or the tested strain, be that a reference strain or a clinical strain isolated from patients. The ARB concentrations that inhibit *E. coli* biofilm could not be found. Based on the above studies, it can be demonstrated that for the discussed etiological factors of biofilm-related infections, the best eliminating phytochemical requiring use in the lowest concentration for *S. aureus* is ARB (0.01 mg/mL); for *E. coli*, it is RT (0.05 mg /mL); and for *P. aeruginosa*, it is GA (0.156 mg/mL), as shown in the graph in Figure 9, which was prepared on the basis of the data contained in Table 2. ARB is a hydroquinone glycoside which, at a concentration of 0.01 mg/mL, has the ability to inhibit *S. aureus* biofilm, hemolytic activity, and the production of extracellular lipase. The mechanism of action is based on the suppression of RNAII expression associated with the detection of the microorganism’s QS. ARB regulates the activity of hemolysins, lipase, exoprotease, enterotoxins, and the resistance of *S. aureus* to methicillin. Therefore, it can be concluded that by inhibiting the expression of RNAII, hydroquinone inhibits the production of virulence factors in *S. aureus*. The compound was effective against both methicillin-sensitive (MSSA) and MRSA strains. Three-dimensional mesh charts measured using confocal laser scanning microscopy and scanning electron bright-field microscopy allowed us to observe reductions in the biomass, its thickness, and the substrate coverage, which reduced by 88%, 89%, and 72%, respectively, compared to controls that were not treated with the phytosubstance at a concentration of 0.001 mg/mL; a concentration of 0.01 mg/mL has been shown to inhibit more than 96% of the biofilm of MSSA strains [74]. At a concentration of 0.05 mg/mL, rutin reduces the viability of the *E. coli* biofilm by 70%, which is related to interfering with QS by reducing the secretion of autoinducer 2 in bacteria while reducing adhesion and damage to bacterial cells, which significantly reduces the damage to cells infected with microorganisms [43]. The anti-biofilm effect against *P. aeruginosa* strains is demonstrated by GA extracted at a concentration of 0.128 mg/mL, which is enhanced further when combined with catechin. When using such a combination, the number of isolates forming a biofilm strongly and moderately decreases from 52.63% to 5.26%. The use of gallic acid against *P. aeruginosa* strains resulted in a significant reduction in the genetic expression of the main pathway regulating QS in the microorganism, i.e., LasI/LasR, in 80% of the tested strains, which indicates that the mechanism of anti-biofilm action of gallic acid involves a reduction in the genetic expression of LasI/LasR. Moreover, gallic acid has been shown to have the ability to eliminate mature *P. aeruginosa* biofilms and microbial biofilms formed from *S. aureus*. GA also has anti-adhesive properties, but also delays the formation of biofilm by bacteria, interferes with the synthesis of AHLs involved in QS, and modifies the hydrophobicity and permeability of the cell membrane. The effect is achieved by increasing the leakage of potassium ions, leading to cell death. It can cause a bactericidal effect in several ways: changing gene expression, disturbing cellular ion homeostasis, and disrupting intracellular signaling [10,42]. Compounds isolated from plants show effectiveness when used in vitro and in vivo on planktonic cells and microbial biofilms. The most common biofilm-related infections include UTI, particularly that associated with *E. coli.* A clinical trial has shown that the use of quercetin at a dose of 500 mg twice daily for 4 weeks in adults improves overall health and reduces the severity of symptoms [75]. This may be related to its anti-adhesive properties against *E. coli* and its ability to limit the biofilm of the microorganism, but can also be attributed to its immunomodulatory effect [75]. A beneficial effect in UTIs associated with E. coli was also obtained in a clinical study in which, after a single intake of 420 mg of arbutin in four volunteers, hydroquinone accumulated in bacterial cells. The hydroquinone concentration in bacterial cells was found at a concentration 20 times higher than that in urine. This allows us to conclude that ARB effectively eradicates bacterial cells closed in a biofilm and that the phytochemicals accumulating in bacterial cells have an inhibitory effect on them. Moreover, the phytochemical does not accumulate in the body and 65% of it is excreted in the form of hydroquinone in urine, which accumulates in bacterial cells and does not affect urine parameters [76]. According to the in vitro MBIC value, ARB is the most effective compound against *S. aureus*, which is a microorganism that permanently colonizes the nasopharynx in over 30% of the world’s population. Colonization is asymptomatic, but in patients with reduced resistance, e.g., hypersensitivity of the respiratory system, it may lead to pneumonia. In randomized double-blind clinical studies, 5.01 mg of ARB contained in pear extract for 4 weeks improved the condition of patients with respiratory hypersensitivity and led to a reduction in the concentration of pro-inflammatory cytokines. The patients’ immunity improved thanks to the increased growth of microorganisms that are part of the natural microflora bacteria. This indicates that ARB could potentially be a supportive element in people with a hyperresponsive respiratory system colonized by *S. aureus.* It could also be used prophylactically due to its ability to limit the activity of virulence factors in the microorganism [38]. No clinical trials involving rutin have been found. However, based on the number and quality of clinical trials for PACs, it can be concluded that they are the most effective therapeutic agent already recommended for the treatment of UTIs complicated by *E. coli*. Their anti-biofilm, anti-adhesive, and anti-inflammatory properties reduce the occurrence of the first episode. This is illustrated by the results pertaining to recurrences of urinary tract infections in randomized clinical trials, in which they were used for at least 6 months in one case, at a dose of 500 mg, and in the other they were used in the form of 125 mL of cranberry juice, which contained 40 mg PACs. The study was carried out accordingly on 182 and 118 patients, where the median age of the second experiment was 50, i.e., this was a group that was naturally more exposed to *E. coli* infection associated with the urinary tract [75,77]. A longer period of use with positive results proved the lack of toxicity resulting from accumulation in the body. Microorganisms’ lack of tolerance to the phytochemical indicates that it was an effective agent in eradicating bacterial biofilm during the duration of the experiment [75]. In a randomized controlled trial of patients with cystic fibrosis complicated by *P. aeruginosa*, after 30 days of using 2400 mg of n-acetylcysteine (NAC) to increase the concentration of VC in the blood, improvement in lung function was achieved. In vitro studies indicate that the MBIC for the vitamin C is 0.156, which is not a high dose. This leads to the conclusion that vitamin C supplementation may have a protective effect on lung tissue and support the treatment of *P. aeruginosa* infection with a bactericidal effect [78]. Similar results were obtained in a randomized controlled trial using VC at a dose of 50 mg for 6 months in children. The result was reduced severity of symptoms in patients with upper respiratory tract symptoms and a bactericidal effect against *P. aeruginosa* infection. VC is an anti-biofilm agent that may be effective against *P. aeruginosa* found in the respiratory tract, but also in wounds [79]. In patients who had undergone tooth extraction, 600 mg of VC improved the healing of wounds, from which *P. aeruginosa* and *S. aureus* are often isolated. The anti-biofilm effect of VC on *P. aeruginosa* is related to the induction of oxidative stress in bacterial cells. This condition leads to the death of bacterial cells with a simultaneous disruption of metabolism in the biofilm. Moreover, VC has a synergistic effect and has been confirmed to allow the use of lower doses of antibiotics, e.g., ceftazidime, in rats, providing a therapeutic effect [80]. According to in vitro studies, the most effective compound for eradicating *P. aeruginosa* biofilm is GA. *P. aeruginosa* is often the etiological factor of pneumonia, in which it forms an exceptionally strong biofilm. It was shown in a randomized controlled trial that the use of 1000 mg of GA in children for 2 months can reduce the frequency of symptoms resulting from this disease. Both *P. aeruginosa* and *S. aureus* have also been isolated in oral infections, and in a randomized clinical trial related to chronic periodontitis disease, *Scrophularia striata* mouthwash was used in adults for 4 weeks to improve health and reduce the severity of symptoms [81]. There is an ongoing need to find alternative treatments for biofilm-related infections and ways to deliver them to target cells. The most effective *S. aureus* biofilm eradication agent, which is potentially effective against strains found in the respiratory tract, is ARB. The plant compound that requires use in the lowest concentration against *E. coli* in order to achieve an anti-biofilm effect is rutin; meanwhile, accounting for available clinical trials, the best compound supporting the treatment of UTIs complicated by *E. coli* is PAC (see Table 3). Clinical trials using phytosubstances to treat diseases associated with the most frequently isolated biofilm-producing microorganisms are shown in Table 3. GA is the most effective factor inhibiting *P. aeruginosa* biofilm in vitro, potentially for strains found in the oral cavity. VC also seems to be a promising treatment for infections involving *P. aeruginosa*.

## 10. Conclusions

Phytochemicals such as quercetin, apigenin, arbutin, proanthocyanidins, vitamin C, gallic acid, and rutin effectively reduce the biofilm of various bacteria. The mechanism behind this effect includes blocking bacterial communication and adhesion. Increased awareness of the potential of phytochemicals may contribute to their wider use in the treatment of infections and tissue regeneration.

Phytochemicals enhance the effects of antibiotics. Combined with antibiotics, they reduce the risk of developing resistance and support treatment. In the future, phytochemicals may become an important element in the fight against multidrug-resistant microorganisms.

The low bioavailability of phytochemicals is problematic. Modern drug delivery systems development, particularly that which involves nanotechnologies, may increase their bioavailability. As research progresses, creating more personalized therapies tailored to individual patients’ needs will become possible.

Both in vitro and clinical studies indicate the great potential of phytochemicals. Further clinical studies are necessary to confirm their safety and effectiveness in antimicrobial therapies, especially in the context of long-term use.

## Figures and Tables

**Table 1:** Bioavailability of phytosubstances for oral intake and skin penetration.

Phytosubstance	Bioavailability [%]		References
QCT (Quercetin glucoside)	Oral Intake	Skin Penetration	
	6.7 (3–17)	17	[23]
AP	30	-	[28]
ARB (Hydroquinone)	-	45	[18]
PAC ((-)epicatechine)	5–10	35	[29]
VC	70–90	95	[30]
GA	70	11	[31]
RT	20	60	[32,33]

AP—apigenin; ARB—arbutin; GA—gallic acid; PAC—proanthocyanidins; QCT—quercetin; RT—rutin; and VC—vitamin C.

**Table 2:** Inhibitory concentrations and forms of phytochemicals for the most frequently isolated biofilm-producing microorganisms.

Phytosubstance	Bacteria Species (Specificity)	Form	Number of Strains	Inhibitory Concentration [mg/mL]		References
				MIC	MBIC	
Quercetin	*S. aureus* (MRSA)	In silico model	ND	0.025	0.02	[24]
	*S. aureus* (mixed biofilm)	EtOH solution	ND	0.064	0.25	[34]
	*E. coli* (ATCC)	EtOH solution	ND	1.0	8.0	[35]
	*P. aeruginosa* (mixed biofilm)	EtOH solution	ND	0.128	0.25	[34]
Apigenin	*S. aureus* (MRSA)	EtOH solution *Hydnum repandum* extract	ND	3.12	9.36	[36]
		Apigenin-7-O-Glucoside	ND	0.28	0.20	[37]
	*E. coli* (ATCC)	EtOH solution *Hydnum repandum* extract	ND	1.56	25	[36]
		Apigenin-7-O-Glucoside	ND	0.14	0.10	[37]
	*P. aeruginosa* (ATCC)	EtOH solution *Hydnum repandum* extract	ND	3.12	15.60	[36]
Arbutin	*S. aureus* (MSSA)	DMSO solution	6538	0.4	0.01	[38]
	*E. coli* (ESBL)	Itxasol©	ND	0.256	-	[38]
	*P. aeruginosa* (clinical strains)	Solution	ND	1.25	2.50	[39]
Proanthocyanidins	*S. aureus* (MRSA)	Root extract *Krameria lappacea*	10	0.064	0.256	[40]
	*E. coli* (tetracycline-resistant)	Cranberry (*Vaccinium macrocarpon)* extract	21	0.1	0.1	[41]
	*P. aeruginosa* (tetracycline-resistant)	Cranberry (*Vaccinium macrocarpon*) extract	21	0.05	0.1	[41]
Vitamin C	*S. aureus* (ATCC)	H_2_O solution	ND	0.15	20	[42]
	*E. coli* (MDR)	H_2_O solution	85	1.25	0.312	[43]
	*P. aeruginosa* (ATCC)	H_2_O solution	50	0.625	0.156	[42]
Gallic acid	*S. aureus* (mixed biofilm)	EtOH solution	ND	2.5	0.5	[34]
	*E. coli* (ATCC)	H_2_O solution	ND	1.71	1.0	[37]
	*P. aeruginosa* (ATCC)	Root extract *Pelargonium hortorum*	10	0.512	0.128	[44]
	*P. aeruginosa* (mixed biofilm)	EtOH solution	ND	2.5	5	[34]
Rutin	*S. aureus* (ATCC)	Solution	22	1.0	0.2	[45]
	*E. coli* O157:H7 (ATCC)	MetOH extract	ND	0.03	0.05	[46]
	*P. aeruginosa* (ESBL, clinical isolate)	MetOH extract *Nepeta curviflora*	ND	10	2.5	[47]

ATCC—American Type Culture Collection; DMSO—dimethyl sulfoxide; ESBL—extended-spectrum beta-lactamase; MIC—Minimal Inhibitory Concentration; MBIC—Minimal Biofilm Inhibitory Concentration; MSSA—methicillin-susceptible *Staphylococcus aureus*; MRSA—methicillin-resistant *Staphylococcus aureus*; ND—no data.

**Table 3:** Clinical trials using phytosubstances to treat diseases associated with the most frequently isolated biofilm-producing microorganisms.

Phytosubstance	Disease	Study Design	Dose (mg per Day)	Duration of Treatment (Weeks)	Patients (Age Mean)	Result	References
QCT	UTI complicated by *Escherichia coli*	CT	500	4	20 (53)	Improved general condition and symptom score; no side effects	[82]
	URTI	Randomized community CT	500 or 1000	12	1002 (51)	Increased efficiency. Reduced URTI severity; reduced number of sick days	[83]
ARB	UTI complicated by *E. coli*	RCT	420	One-time intake	4 (ND)	Accumulation of hydroquinone in bacterial cells	[76]
	Respiratory hypersensitivity	Randomized double-blind clinical studies	5.01	4	20 (ND)	Growth of physiological bacterial microflora stimulation; activity of pro-inflammatory cytokines reduction	[84]
PAC	UTI complicated by *E. coli*	RCT	40	24	118 (50)	Reduced UTI recurrences	[77]
	UTI complicated by *E. coli*	RCT	500	24	182 (ND)	Reduced recurrences and the first episode of UTI occurrence	[75]
VC	Acute inflammation induced by *E. coli* LPS	Balanced, placebo-controlled cross-over study	320 or 480 per kg	One-time intake	36 (ND)	Abolished effects of LPS secreted by *E. coli* (restored response to Ach, dilated blood vessels)	[85]
	CF complicated by *Pseudomonas aeruginosa*	RCT	2400 NACs which increase VC content	4	21(ND)	Improved lung function	[78]
	URTI	RCT	50 together with probiotic	24	171 (6)	Reduced severity of symptoms	[86]
	Wound healing after dental extraction	RCT	600 mg one-time intake	3	30 (ND)	Improved soft tissue healing	[79]
GA	Vaginitis	RCT	0.276	1	120 (36)	Therapeutic effect on mixed vaginitis; reduced pain; eliminated symptoms	[87]
	URTI and digestive system	RCT	7.8	8	ND (7)	Reduced symptoms’ frequency; improved blood test results	[88]

AP—apigenin; ARB—arbutin; CF—cystic fibrosis; CT—clinical trial; GA—gallic acid; LPS—lipopolysaccharide; NAC—n-acetylcysteine; ND—no data; PAC—proanthocyanidins; QCT—quercetin; RT—rutin; RCT—randomized control trial; URTI—upper respiratory tract infection; VC—vitamin C.

## Data Availability

No new data were created or analyzed in this study. Data sharing is not applicable to this article.
